# Pediatric MR cholangiopancreatography: comparison of 3D fast spin-echo and single-shot fast spin-echo thick-slab techniques for pancreaticobiliary anatomy

**DOI:** 10.55730/1300-0144.6222

**Published:** 2026-04-30

**Authors:** Seda KAYNAK ŞAHAP, Suat FİTOZ

**Affiliations:** Division of Pediatric Radiology, Department of Radiology, Faculty of Medicine, Ankara University, Ankara, Turkiye

**Keywords:** Magnetic resonance cholangiopancreatography, pediatric, pancreaticobiliary anatomy, image quality

## Abstract

**Background/aim:**

This study compared thick-slab and three-dimensional (3D) T2-weighted magnetic resonance cholangiopancreatography (MRCP) sequences in pediatric patients with respect to image quality and visualization of the pancreaticobiliary anatomy.

**Materials and methods:**

The MRCP examinations of 62 pediatric patients evaluated for suspected pancreaticobiliary pathology were retrospectively reviewed, comprising a total of 69 studies. Thick-slab single-shot fast spin-echo and 3D fast spin-echo MRCP sequences were compared. The visualization of pancreaticobiliary structures was assessed using a qualitative visual scoring system. Image quality was graded as good, moderate, poor, or nondiagnostic. Biliary anatomical variations were also evaluated. Statistical analysis was performed using appropriate nonparametric tests with a significance threshold of p < 0.05.

**Results:**

The most common clinical indication for MRCP was screening for primary sclerosing cholangitis. Overall image quality did not differ significantly between thick-slab and 3D MRCP sequences. However, 3D MRCP demonstrated significantly superior visualization of the right and left proximal bile ducts, the cystic duct, and the pancreatic duct in the pancreatic tail compared to thick-slab sequences. Biliary anatomical variations were identified in 29% of patients, with type 3a being the most frequent variation.

**Conclusion:**

Compared to thick-slab MRCP, 3D MRCP provides improved visualization of the pancreaticobiliary anatomy in children. Optimized 3D MRCP protocols may enhance diagnostic confidence in pediatric pancreaticobiliary imaging.

## 1. Introduction

Magnetic resonance cholangiopancreatography (MRCP) is a noninvasive imaging modality widely used for the evaluation of the pancreaticobiliary anatomy and various pathologies. However, obtaining diagnostically adequate MRCP images in children remains challenging because of the small diameters of pediatric bile ducts, motion artifacts, limited patient cooperation, and difficulties with breath-holding, all of which may compromise image quality [1,2].

Accurate depiction of normal pancreaticobiliary anatomy is fundamental for reliable identification of pathological findings. Therefore, optimization of MRCP techniques is particularly important in pediatric imaging [1–3]. Thick-slab and three-dimensional (3D) heavily T2-weighted MRCP sequences are commonly used in clinical practice, each with distinct advantages and limitations. When 3D T2-weighted MRCP images are acquired without significant motion artifacts, the method provides higher signal-to-noise ratio (SNR) and improved anatomical detail through volumetric acquisition; however, respiratory misregistration may substantially degrade image quality in children with irregular breathing. In contrast, thick-slab MRCP is obtained in a single breath-hold, minimizing respiratory misregistration and providing a rapid overview of the pancreaticobiliary tree, albeit with potential limitations related to image blurring, flow artifacts, and in-plane volume averaging [4,5].

Previous studies [5–7] comparing thick-slab and 3D MRCP have predominantly focused on adult populations and reported inconsistent results, while data from pediatric patients remain limited. Accordingly, the aim of this study was to compare thick-slab and 3D MRCP sequences in pediatric patients, with particular emphasis on image quality and visualization of the pancreaticobiliary anatomy, in order to better define their relative diagnostic value in routine clinical practice.

## 2. Materials and methods

### 2.1. Patient population and magnetic resonance imaging protocol

MRCP images from 62 consecutive patients who underwent evaluation for suspected pancreaticobiliary system pathology during a defined study period (2011–2015) were retrospectively reviewed. The cohort included 35 girls and 27 boys with an age range of 4 days to 17 years. Seven patients underwent more than one examination, resulting in a total of 69 MRCP examinations. Inclusion criteria consisted of patients under 18 years of age who underwent MRCP for evaluation of suspected pancreaticobiliary pathology during the study period. Patients aged 18 years or older and cases with incomplete imaging data were excluded. For the comparative image quality analysis between the two MRCP techniques, patients with biliary atresia and cases in which only one imaging sequence was available were excluded, as anatomical comparison was not feasible in these situations.

Ethical approval was obtained from the local ethics committee (approval no: 08-340-15) and informed consent was not required due to the retrospective nature of the study. MRCP examinations were performed for a variety of clinical indications, most commonly for screening of primary sclerosing cholangitis in patients with inflammatory bowel disease (n = 25), followed by suspected pancreatitis (n = 13). Other indications included chronic liver disease (n = 7), anastomotic strictures in patients with hepatoportoenterostomy (n = 6), congenital biliary dilatation (n = 5), suspected cholelithiasis or choledocholithiasis (n = 5), biliary atresia (n = 3), assessment of cholestasis (n = 2), suspicion of biloma (n = 2), and prediagnosis of double gallbladder (n = 1).

All patients were screened for well-known contraindications to magnetic resonance (MR), and MRCP examinations were performed after a fasting period of at least 4–6 h. General anesthesia was administered in 31 cases due to inability to hold breath, lack of cooperation, or underlying neurodevelopmental conditions.

The MR examinations of 46 patients were performed using a 1.5-T MR scanner (Optima MR 450 W, GE HealthCare, Chicago, IL, USA) with an eight-channel cardiac coil. Following the acquisition of three-plane localizer images, axial T2-weighted PROPELLER sequences were obtained using respiratory triggering, followed by coronal single-shot T2 single-shot fast spin-echo sequences.

High-resolution 3D T2-weighted images were then acquired using a multishot technique with respiratory triggering in the axial plane (covering the common bile duct from the hepatic bifurcation to the pancreatic duct termination) and in the coronal oblique plane (including the pancreatic duct).

T1-weighted images were obtained during approximately 10-s breath-hold periods. Additionally, coronal thick-slab images of approximately 4 cm in thickness were acquired during breath-hold periods of 2–3 s, centered on the confluence of the common bile duct and the main pancreatic duct, with an average angulation of 15° to 20°. Minimum intensity projection (MIP) images were generated in all three planes from the source images.

Field-of-view values were adjusted according to the size of the child and ranged between 25 and 40 cm. Slice thickness ranged from approximately 1–1.5 mm for 3D acquisitions to up to 40 mm for thick-slab images. Matrix sizes were also comparable between systems, generally ranging from approximately 288 × 192 to 384 × 256, and echo times were within similar ranges across both scanners.

MR examinations of the remaining 23 patients were performed with another 1.5-T scanner (Ingenia MR, Philips Healthcare, Orlando, FL, USA) using a similar MRCP protocol. Although the imaging protocols were not identical, acquisition parameters were highly similar and kept as comparable as possible across the two MR systems.

### 2.2. Image analysis

The comparative analysis of thick-slab and 3D MRCP sequences was performed on an examination basis. Five examinations were excluded from the comparative analysis: three due to biliary atresia, in which anatomical distortion precluded reliable comparison, and two due to the availability of only one MRCP sequence. Consequently, 64 MRCP examinations were included in the final image quality assessment.

Image evaluation was performed independently by a pediatric radiologist with 16 years of experience and a radiology resident. Both readers were blinded to the clinical information and final diagnosis. The visibility of pancreaticobiliary structures was assessed using a predefined qualitative scoring system. In cases of discrepancy between the two readers, a consensus was reached through joint review.

On both 3D MRCP and radial thick-slab images the visibility of the right and left proximal bile ducts (sectoral branches extending to the first branching point were considered proximal bile ducts), right and left main hepatic ducts, main hepatic duct, cystic duct, gallbladder, common bile duct, pancreatic duct at the level of head and tail, and minor pancreatic duct were scored and then evaluated in terms of the presence of dilatation and intraluminal pathologies. In addition, visual scoring was performed individually for thick-slab and 3D images in terms of quality of image, and the images were scored as good (3 points), moderate (2), poor (1), or nondiagnostic (0) according to the visibility of the pancreaticobiliary anatomy. Poor and moderate image quality categories were combined to simplify statistical analysis and improve interpretability.

Image quality and anatomical visualization were evaluated using qualitative scoring, reflecting routine clinical practice in pediatric MRCP. Quantitative image quality metrics such as SNR and contrast-to-noise ratio (CNR) were not assessed due to the study’s retrospective design and variability in acquisition parameters across examinations.

The patients were also evaluated for the presence of anomalous pancreaticobiliary junction (APBJ) and concomitant pathologies. APBJ was considered to be the common bile duct and main pancreatic duct joining at a distance of more than 5 mm from the duodenum.

Moreover, the presence of biliary variations according to the classification recommended by Choi et al. was noted [8]. Cystic duct insertion variants were also evaluated, with distal one-third common bile duct insertions defined as inferior and left-sided main hepatic duct insertions defined as medial.

### 2.3. Statistical analysis

Statistical analysis was performed using IBM SPSS Statistics 22.0 for Windows (IBM Corp., Armonk, NY, USA). Descriptive statistics were expressed as mean ± standard deviation, frequency, and percentage values.

Comparisons between 3D MRCP and thick-slab MRCP were performed using paired statistical analyses, as both techniques were evaluated within the same examinations. Categorical variables were analyzed using the McNemar or McNemar–Bowker test as appropriate.

Subgroup analysis was performed to evaluate the potential effect of general anesthesia on image quality. Patients were categorized into anesthetized and nonanesthetized groups, and comparisons were performed separately for thick-slab and 3D MRCP sequences using the chi-square test.

The statistical analysis was primarily exploratory and focused on identifying clinically relevant differences between imaging techniques rather than estimating effect sizes or performing multiple comparison corrections. Values of p < 0.05 were considered statistically significant.

## 3. Results

The study population consisted of 62 patients (35 girls, 56.5%; 27 boys, 43.5%). Of these patients, 12.9% (n = 8) were ≤1 year of age, 14.6% (n = 9) were 2–5 years, 41.9% (n = 26) were 6–14 years, and 30.6% (n = 19) were ≥15 years. The youngest patient was 4 days old and the oldest patient was 17 years old.

The most common indication for MRCP was screening for primary sclerosing cholangitis, which accounted for 36.2% (n = 25) of all examinations.

Pathological findings were identified in more than half of the MRCP examinations (52.2%, n = 36), while the remaining 47.8% (n = 33) were normal. Notably, all MRCP studies performed for suspected biliary atresia, choledochal cyst, or cholelithiasis demonstrated pathological findings. A high rate of pathology was also observed in examinations performed for suspected pancreatitis, with 84.6% (n = 11) yielding abnormal results. In contrast, MRCP results obtained for the screening of primary sclerosing cholangitis were predominantly normal, with 96% (n = 24) showing no pathological findings and only 4% (n = 1) demonstrating abnormalities.

Visual image quality for thick-slab MRCP was rated as poor for 3 patients, moderate for 12, and good for 49, while for 3D MRCP the visual image quality was poor for 4 patients, moderate for 11, and good for 49. Due to the small number of poor-quality images, the poor and moderate groups were combined for analysis. No significant differences in image quality scores were observed between the age groups. Although overall image quality scores were comparable between 3D MRCP and thick-slab MRCP, segment-specific evaluation revealed that 3D MRCP provided better visualization of certain pancreaticobiliary structures.

**Figure 1:**
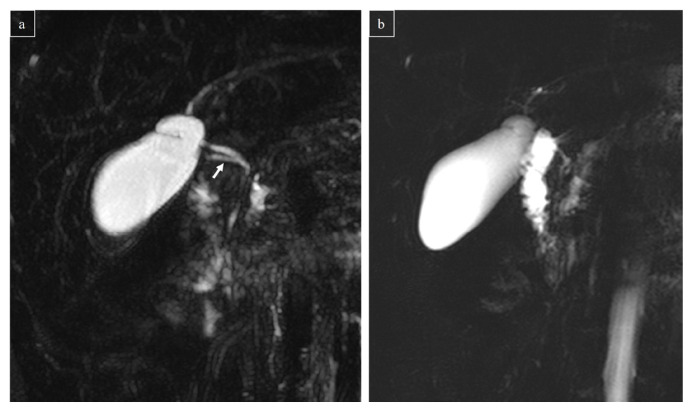
The cystic duct (arrow) is clearly visualized on the 3D MRCP image **(a)**, whereas it is not visible on the thick-slab MRCP image **(b)** due to superimposition by the duodenum.

This study reveals that 3D MRCP (Figure 1a) provides superior visualization of the pancreaticobiliary anatomy compared to thick-slab imaging (Figure 1b). The obtained 3D images provided a significantly better depiction of the right and left proximal bile ducts, the cystic duct, and the pancreatic duct in the tail of the pancreas (p < 0.05) (Table 1). Notably, the minor pancreatic duct was visualized for 4 of 62 patients, and in 3 of these cases it was only identifiable on 3D images, remaining indistinguishable on thick-slab images. The right proximal bile duct could not be visualized for any patient aged ≤1 year on either sequence, highlighting the limitations of imaging very young patients.

Subgroup analysis based on anesthesia status demonstrated no statistically significant difference in image quality between anesthetized and nonanesthetized patients for either thick-slab MRCP (p = 0.567) or 3D MRCP (p = 0.252). The distribution of image quality scores was comparable between the two groups in both sequences.

**Figure 2:**
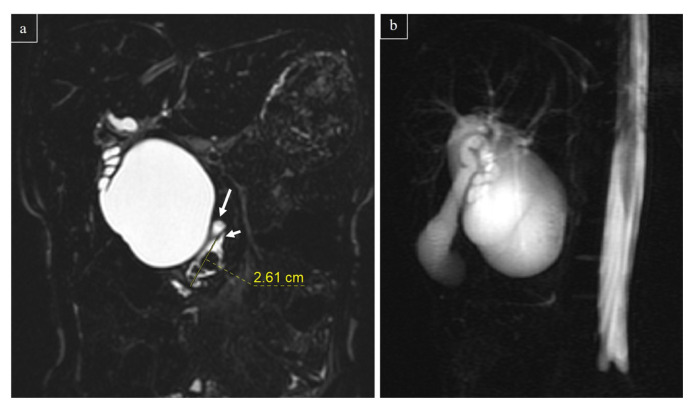
The 3D MRCP image **(a)** demonstrates the main bile duct (long arrow) and pancreatic duct (short arrow) joining at a distance from the duodenum, consistent with anomalous pancreaticobiliary junction (APBJ). In contrast, the thick-slab MRCP image **(b)** fails to demonstrate an APBJ due to the large size of the choledochal cyst.

An APBJ was identified in two cases, including one patient with a type 4a choledochal cyst and one patient with groove pancreatitis (Figures 2a and 2b).

**Figure 3:**
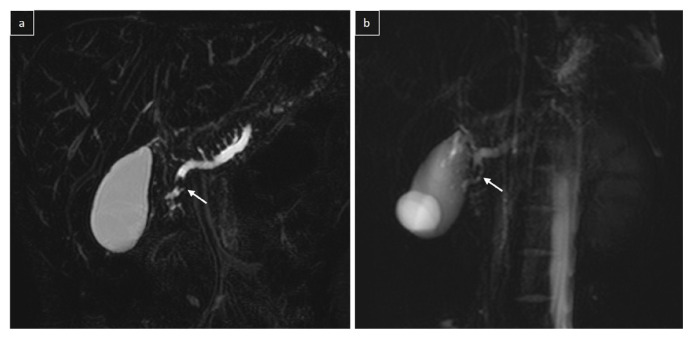
The 3D MRCP image **(a)** and thick-slab MRCP image **(b)** demonstrate a filling defect (arrows) at the neck of the dilated pancreatic duct, initially interpreted as a stone. However, subsequent ERCP revealed no stone and the filling defect was attributed to air bubbles.

In one patient evaluated for pancreatitis, both 3D MRCP and thick-slab imaging successfully demonstrated a filling defect in the main pancreatic duct. However, it was initially interpreted as a stone on MRCP and was subsequently shown to represent an air bubble on endoscopic retrograde cholangiopancreatography (ERCP) (Figures 3a and 3b).

**Figure 4:**
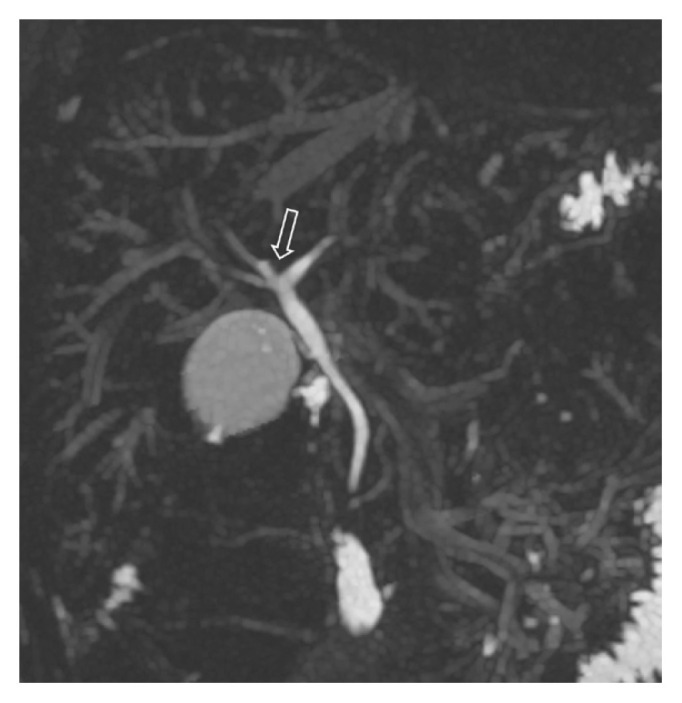
Maximum intensity projection of the 3D MRCP image demonstrates trifurcation of the biliary ducts, consistent with a type 2 biliary anatomical variation (arrow).

Biliary anatomical variations were observed in 18 of the 62 patients (29%), with two patients exhibiting multiple variations (Figure 4). The types and frequencies of these variations are summarized in Table 2.

## 4. Discussion

In this study, we compared thick-slab and 3D MRCP sequences in a pediatric population and demonstrated that 3D MRCP provides superior visualization of the pancreaticobiliary anatomy, particularly for smaller and more tortuous ductal structures. Despite the inherent challenges of pediatric MR imaging, including the small diameter of the ducts, motion artifacts, and limited breath-holding capability, 3D MRCP showed improved anatomical detail compared to thick-slab imaging. These findings underscore the importance of optimized MRCP protocols for children, where accurate depiction of normal anatomy is essential for confident identification of pathological changes. This is consistent with large pediatric series reporting that MRCP facilitates reliable, noninvasive evaluation of the pancreaticobiliary anatomy and has a significant impact on clinical decision-making [9].

Previous studies [5–7] conducted in adults reported heterogeneous results, with some demonstrating superior anatomical depiction using thick-slab MRCP sequences and others favoring 3D MRCP techniques, while direct comparisons between 3D and thick-slab MRCP techniques in pediatric populations remain limited. While previous findings [4] have indicated that thick-slab MRCP sequences may be advantageous for the assessment of small, distal bile ducts, our findings suggest that 3D MRCP provides significantly superior visualization of the right and left proximal bile ducts, the cystic duct, and the pancreatic duct in the pancreatic tail. This advantage likely reflects the multiplanar reconstruction capability of 3D MRCP and its improved depiction of ductal continuity, in addition to reduced susceptibility to partial volume effects from adjacent gastric fluid, which may limit visualization of the pancreatic duct in the tail on thick-slab MRCP [6,10–12]. Visualization of small-caliber ducts remains a known challenge in pediatric MRCP. In our study, the right proximal bile duct could not be visualized in patients aged 1 year or younger, which may be related to the very small diameter of the ducts in infants, making reliable evaluation technically challenging.

It should be noted that a higher proportion of MRCP examinations in our cohort were performed under general anesthesia compared to prior pediatric studies, which may have reduced respiratory irregularity and motion artifacts related to breath-holding, thereby potentially attenuating the known limitations of 3D MRCP. General anesthesia is known to reduce respiratory motion and associated motion artifacts, and it may theoretically improve MR image quality, particularly in pediatric populations. Therefore, it may be considered a potential confounding factor when comparing MRCP techniques. However, in our study, subgroup analysis demonstrated that image quality did not significantly differ between anesthetized and nonanesthetized patients for either thick-slab or 3D MRCP. This finding suggests that the superior visualization of pancreaticobiliary structures observed with 3D MRCP cannot be solely attributed to anesthesia-related motion reduction, but rather reflects intrinsic technical advantages of the 3D acquisition.

Detection of APBJ is clinically important, and although ERCP has traditionally been considered the reference standard, MRCP has increasingly been proposed as a noninvasive alternative [13–16]. While early studies reported limited sensitivity of MRCP, subsequent investigations using optimized techniques demonstrated improved detection rates, particularly in adults [16–18]. In pediatric populations, diagnostic limitations have been attributed to young age and large cyst size [16,18]. In our cohort, MRCP findings in patients with choledochal cysts were largely concordant with surgical and pathological results; however, APBJ visualization remained limited in very young patients with large cysts, consistent with prior reports.

MRCP plays an established role in the evaluation of biliary pathology, including choledocholithiasis; however, several limitations related to image interpretation should be recognized [19–21]. Filling defects observed on MRCP are not specific for stones and may be mimicked by air bubbles, blood clots, dense bile content, flow-related signal voids, metallic stents, or susceptibility artifacts [22]. In the presence of markedly dense bile, both 3D and thick-slab MRCP images may be limited, whereas T1-weighted imaging provides important complementary information by facilitating more confident differentiation of true filling defects [23]. In addition, extrinsic vascular compression most commonly involving the common hepatic duct or left hepatic duct due to the course of the right hepatic artery may mimic ductal filling defects or strictures [24]. In one of our cases, an apparent filling defect in the common hepatic duct was attributed to arterial compression, which could only be confidently identified on 3D images. Although thick-slab MRCP may obscure small filling defects or subtle strictures due to partial volume effects, similar limitations may also be encountered on MIP reconstructions. Therefore, careful correlation with thin-section images remains essential to avoid misinterpretation and inaccurate assessment of ductal pathology [25,26].

While biliary anatomical variations have been well described in adult MRCP studies, systematic data in pediatric MRCP studies are scarce. Nevertheless, MRCP provides a noninvasive means of delineating biliary anatomy and identifying variations in children, particularly in preoperative and transplant settings. In the present study, type 3a biliary anatomy defined by drainage of the right posterior sectoral duct into the left main hepatic duct was the most frequently observed, similar to previous findings reported in both adult and pediatric MRCP studies [8,27,28]. The anatomical variations identified in this study may have clinical relevance, particularly in surgical planning and interventional procedures, where accurate delineation of the biliary anatomy is essential.

This study has several limitations. Its retrospective design and relatively small sample size may limit the generalizability. The heterogeneous spectrum of pancreaticobiliary pathologies resulted in small subgroup sizes, precluding detailed subgroup analyses. Furthermore, different underlying pathologies may have influenced duct visualization to varying degrees, potentially affecting the overall results. In addition, the predominance of screening indications in a substantial proportion of patients may have influenced the overall visualization outcomes. The use of two different MR systems may have introduced variability in image quality despite efforts to standardize acquisition parameters, and this constitutes a limitation of the study.

Image assessment was based on qualitative visual scoring rather than quantitative metrics such as SNR or CNR, and interobserver agreement was not assessed. However, evaluations were performed independently by two readers followed by consensus review, reflecting routine clinical practice. In addition, effect size estimation and correction for multiple comparisons were not performed, which may have limited the statistical robustness of the findings.

Moreover, distal ductal branches were not specifically included in the scoring system, which may limit comparisons with some prior studies. However, this is unlikely to be clinically significant, as assessment of the proximal ducts and overall ductal continuity is generally more relevant in pediatric practice. The absence of a consistent reference standard, such as ERCP or surgical findings, may limit the assessment of diagnostic accuracy; however, ERCP is invasive and not technically feasible in children and it has well-known risks of radiation.

In conclusion, our findings indicate that 3D T2-weighted MRCP offers improved visualization of the pancreaticobiliary anatomy in children compared to thick-slab imaging, particularly for proximal intrahepatic ducts, the cystic duct, and the pancreatic duct in the pancreatic tail. Optimized 3D MRCP protocols enhance the depiction of ductal continuity; however, attention to potential pitfalls and the use of complementary sequences remain essential for accurate interpretation.

## Figures and Tables

**Table 1:** Comparison of pancreaticobiliary anatomy between 3D MRCP and thick-slab MRCP.

Pancreaticobiliary segment	3D MRCP visibility	Thick-slab, not visible, n (%)	Thick-slab, visible, n (%)	p
Right proximal bile duct	Not visible (n = 10)	8 (80.0)	2 (20.0)	<0.001
	Visible (n = 54)	19 (35.2)	35 (64.8)	
Left proximal bile duct	Not visible (n = 14)	13 (92.9)	1 (7.1)	<0.001
	Visible (n = 50)	21 (42.0)	29 (58.0)	
Cystic duct	Not visible (n = 10)	13 (76.5)	4 (23.5)	0.019
	Visible (n = 54)	15 (31.9)	32 (68.1)	
Pancreatic duct (tail)	Not visible (n = 10)	16 (88.9)	2 (11.1)	0.013
	Visible (n = 54)	12 (26.1)	34 (73.9)	

Data are presented as number (percentage). Visualization was categorized based on three-dimensional (3D) magnetic resonance cholangiopancreatography (MRCP) findings. Comparisons between 3D MRCP and thick-slab MRCP were performed using paired statistical analysis.

**Table 2:** Distribution of biliary anatomical variations detected on MRCP.

Biliary anatomical variation	Classification	Frequency, % (n)
Right posterior sectoral duct draining into the left main hepatic duct	Type 3a	12.9% (8)
Trifurcation of the right anterior, right posterior, and left main hepatic ducts	Type 2	6.5% (4)
Right posterior sectoral duct draining into the cystic duct	Type 3c	1.6% (1)
Right posterior sectoral duct draining into the common hepatic duct just proximal to the cystic duct insertion	Type 3b	1.6% (1)
Right posterior sectoral duct draining into the common bile duct distal to the cystic duct insertion	Type 7	1.6% (1)
Aberrant bile duct draining segment VI into the common hepatic duct	Type 5a	1.6% (1)
Cystic duct draining into the left side of the common hepatic duct (medial insertion)	—	4.8% (3)
Cystic duct draining into the distal one-third of the common bile duct (low insertion)	—	1.6% (1)

Percentages were calculated based on the total number of patients (n = 62). Classification types were based on established biliary anatomy classifications; cystic duct variants without a defined classification were evaluated descriptively.
